# Comment on: Point-of-care testing in community pharmacies to improve antimicrobial stewardship in respiratory infections: a scoping review of effectiveness, implementation and cost-effectiveness

**DOI:** 10.1093/jacamr/dlag220

**Published:** 2026-10-12

**Authors:** Abdulrasheed Ibrahim

**Affiliations:** Department of Surgery, Ahmadu Bello University (ABU) & Ahmadu Bello University Teaching Hospital (ABUTH), Shika-Zaria, Kaduna, Nigeria

To the Editor,

I read with interest the scoping review by Saha *et al.*^1^ on point-of-care testing (POCT) in community pharmacies to improve antimicrobial stewardship for respiratory tract infections. The authors offer a valuable synthesis, mapping implementation determinants against the Consolidated Framework for Implementation Research. They confirm that C-reactive protein (CRP) POCT can meaningfully reduce unnecessary antibiotic supply. Across the pooled CRP-POCT studies, roughly four in five patients had a CRP below 20 mg/L, indicating no need for antibiotics.^1^ I wish to extend two points that bear directly on whether these conclusions translate to the settings where the antimicrobial resistance (AMR) burden is heaviest.

The authors themselves note a decisive limitation: all but one of the 27 included studies were conducted in high-income countries.^1^ They rightly call for contextual, multifaceted implementation strategies and further trials in both high- and low-income settings.^1^ I argue that in the highest-burden settings this requires more than adaptation within the formal pharmacy sector: it requires rethinking both who delivers frontline care and whether the diagnostic exists at all. This matters because low- and middle-income countries (LMICs) bear the greatest share of AMR-associated mortality.^2^

First, on implementation and behaviour change. The facilitators the review identifies presuppose a regulated, formally trained workforce: professional pharmacy structures, quality-assurance systems and reimbursement mechanisms. In rural sub-Saharan Africa and South Asia, the first and often only point of contact is an informal provider: a drug seller, patent-medicine vendor or ‘village doctor’. These providers operate outside professional registration and stewardship governance. Non-prescription and informal antibiotic supply is pervasive in these settings.^3^ Behaviour-change theory reminds us that prescribing is governed not only by knowledge and motivation but by capability and opportunity.^4,5^ Importing CRP-POCT together with strategies validated among community pharmacists will not straightforwardly reshape the reflexive dispensing habits of providers embedded in different incentive and regulatory structures. Reducing unnecessary antibiotic supply in these networks, a form of de-implementation, will require behavioural models developed *for*, and *with*, informal providers rather than adapted *post hoc* from high-income templates.

Second, on diagnostic stewardship. The review’s optimism rests on settings where the diagnostic itself is available, quality-assured and affordable. Encouragingly, landmark trials in Vietnam and in Thailand and Myanmar have shown that CRP-POCT can safely reduce antibiotic prescribing in LMIC primary care.^6,7^ Yet these were resourced research environments within the formal sector. In the informal settings that deliver the bulk of frontline care, low-cost point-of-care diagnostics are largely absent. There is little supporting infrastructure for device calibration, quality control or result interpretation. A behavioural intervention cannot substitute for the capability to distinguish self-limiting viral illness from bacterial infection at the point of care. Diagnostic stewardship ensures that the appropriate test is both available and correctly used. In these contexts, it is therefore not an adjunct to antimicrobial stewardship but a precondition for it.^8^

I suggest that future reviews, and more importantly future implementation research, stratify findings by economy and by provider type. The near-total absence of LMIC and informal-sector evidence that Saha *et al.*^1^ helpfully identify deserves to be a priority for future research. Building on their call, embedding CRP-POCT within context-specific behavioural frameworks and functional diagnostic infrastructure, as envisaged in World Health Organization guidance for stewardship in LMICs, offers a more realistic path.^9^ That is preferable to assuming that interventions effective in high-income community pharmacies will transfer intact to the frontline of the AMR crisis.
